# The insulin-like growth factor system in multiple myeloma: diagnostic and therapeutic potential

**DOI:** 10.18632/oncotarget.8982

**Published:** 2016-04-25

**Authors:** Liesbeth Bieghs, Hans E. Johnsen, Ken Maes, Eline Menu, Els Van Valckenborgh, Michael T. Overgaard, Mette Nyegaard, Cheryl A. Conover, Karin Vanderkerken, Elke De Bruyne

**Affiliations:** ^1^ Department of Hematology and Immunology, Myeloma Center Brussels, Vrije Universiteit Brussel, Brussels, Belgium; ^2^ Department of Hematology, Aalborg Hospital, Aalborg University, Denmark; ^3^ Department of Biomedicin, Aarhus University, Aarhus, Denmark; ^4^ Clinical Cancer Research Center, Aalborg University Hospital, Denmark; ^5^ Department of Clinical Medicine, Aalborg University, Denmark; ^6^ Department of Chemistry and Biotechnology, Aalborg University, Denmark; ^7^ Division of Endocrinology, Metabolism and Nutrition, Endocrine Research Unit, Mayo Clinic, Rochester, NY, USA

**Keywords:** multiple myeloma, Insulin-like growth factor, IGF-I targeting

## Abstract

Multiple myeloma (MM) is a highly heterogeneous plasma cell malignancy. The MM cells reside in the bone marrow (BM), where reciprocal interactions with the BM niche foster MM cell survival, proliferation, and drug resistance. As in most cancers, the insulin-like growth factor (IGF) system has been demonstrated to play a key role in the pathogenesis of MM. The IGF system consists of IGF ligands, IGF receptors, IGF binding proteins (IGFBPs), and IGFBP proteases and contributes not only to the survival, proliferation, and homing of MM cells, but also MM-associated angiogenesis and osteolysis. Furthermore, increased IGF-I receptor (IGF-IR) expression on MM cells correlates with a poor prognosis in MM patients. Despite the prominent role of the IGF system in MM, strategies targeting the IGF-IR using blocking antibodies or small molecule inhibitors have failed to translate into the clinic. However, increasing preclinical evidence indicates that IGF-I is also involved in the development of drug resistance against current standard-of-care agents against MM, including proteasome inhibitors, immunomodulatory agents, and corticoids. IGF-IR targeting has been able to overcome or revert this drug resistance in animal models, enhancing the efficacy of standard-of-care agents. This finding has generated renewed interest in the therapeutic potential of IGF-I targeting in MM. The present review provides an update of the impact of the different IGF system components in MM and discusses the diagnostic and therapeutic potentials.

## INTRODUCTION

Multiple myeloma (MM) is a plasma cell (PC) malignancy characterized by the proliferation of malignant monoclonal PCs in the bone marrow (BM). MM is the second most diagnosed hematological malignancy, accounting for 1% of all new cancer cases annually [1]. The incidence of MM is more common in men and increases with age. In 2012, the number of new cases of myeloma was 6.3 per 100,000 individuals in the United States [2]. The incidence rate in Europe the same year was approximately 3.9 [3]. Clinical features of MM include hypercalcemia, renal failure, anemia, and bone disease (frequently referred to by the acronym CRAB), which represent evidence of end-organ failure [4]. MM is consistently preceded by an asymptomatic premalignant disease known as monoclonal gammopathy of undetermined significance (MGUS) [5]. MGUS is characterized by the presence of a serum monoclonal protein (M protein, less than 3 g/dl) and the BM containing less than 10% monoclonal PCs without any evidence of end-organ damage [6]. MM is a genetically highly heterogeneous disease with the lack of a universal driver mutation and the presence of a high number of non-recurrent genetic defects [7–9]. Common primary defects thought to be the initiating events in MM development are broadly classified into two categories: hyperdiploid (multiple trisomies) and non-hyperdiploid genetic defects. Frequently observed hyperdiploid defects are trisomies of chromosomes 3, 5, 7, 9, 11, 15, 19, or 21. Non-hyperdiploid defects are mostly monosomy 13 or translocations involving the immunoglobulin heavy chain (IgH) locus (14q32.3). Recurrent translocation partners for this locus are t(4;14), t(6;14), t(11;14), t(14;16), and t(14;20), affecting FGFR3/MMSET, CCND (cyclin D family) genes, and MAF genes [10]. These primary defects are present in both MGUS and MM cells and uniformly lead to the overexpression of CCND genes, resulting in deregulation of the G_1_/S cell cycle transition point, a key early molecular abnormality in myeloma [11]. Progression of MGUS to MM is associated with secondary genetic aberrations, such as mutations in KRAS, BRAF, and NRAS, p53 mutations, and upregulation of Myc and NF-κB [12–14]. However, overwhelming evidence indicates that these genetic defects alone are insufficient to induce progression from MGUS to MM, as a permissive BM microenvironment is required for the emergence of overt MM. In this BM microenvironment, MM cells interact closely with hematopoietic and non-hematopoietic cells, (including mesenchymal stromal cells (MSCs), osteoblasts, osteoclasts, and immune cells) through adhesion molecules and the production of different growth factors, such as insulin-like growth factor (IGF), interleukin-6 (IL-6), VEGF, a proliferation-inducing ligand (APRIL), B cell activating factor (BAFF), and stromal cell-derived factor-1α (SDF-1α). These bidirectional interactions support the survival, proliferation, and homing of MM cells and contribute to drug resistance. Moreover, these interactions result in osteolysis, increased angiogenesis, and immune suppression [15–17].

Over the last decade, the introduction of agents targeting both MM cells and interactions with the BM niche, such as proteasome inhibitors (PIs; e.g., bortezomib and carfilzomib) and immunomodulatory agents (IMiDs; e.g., thalidomide, lenalidomide, and pomalidomide), has significantly prolonged the survival of MM patients [18, 19]. In 2015, front-line treatment of young and fit MM patients (under 65 years of age) consisted of induction therapy with different combinations of PIs together with IMiDs, corticosteroids (mostly dexamethasone), and/or anti-mitotic agents (e.g., cyclophosphamide or doxorubicin) to reduce tumor burden. The induction therapy is followed by a stem cell harvest, high-dose melphalan treatment, and autologous stem cell transplantation (ASCT) [20]. After ASCT, consolidation (short-term) and/or maintenance (long-term) therapy are used to improve responses after induction therapy and increase time of remission after successful induction therapy [21]. Both consolidation and maintenance therapy consist mainly of PI- or IMiD-based regimens and are beneficial for progression-free survival, and possibly also overall survival [22–24]. For patients ineligible for transplantation, bortezomib-melphalan-prednisone or bortezomib-prednisone-thalidomide combination regimens are the standard-of-care options [25]. In addition, patients receive supportive therapy for the management of hypercalcemia, skeletal complications, anemia, infections, and pain [26]. However, despite the major advances in MM therapy, MM is often incurable, with an overall survival of 5.2 years after diagnosis depending on age and therapy [18, 27, 28]. A major problem in MM treatment is the development of drug resistance against current standard-of–care agents [29]. This highlights the need to enhance our understanding of MM biology and the occurrence of drug resistance, and to identify novel targets and treatment options. The IGF system plays a pivotal role in MM tumor biology [30]. Freund *et al.* were the first to demonstrate that IGF-I is an important growth factor for MM cells [31]. Since that study, we and others have demonstrated multiple roles of the IGF system in the pathogenesis of MM, and various strategies targeting the IGF system have been evaluated in clinical trials [32–34]. In this review we summarize the different components of the IGF system and discuss their contribution to MM development. Moreover, we will also discuss the diagnostic and therapeutic potential in MM.

## THE IGF SYSTEM

**Figure 1:**
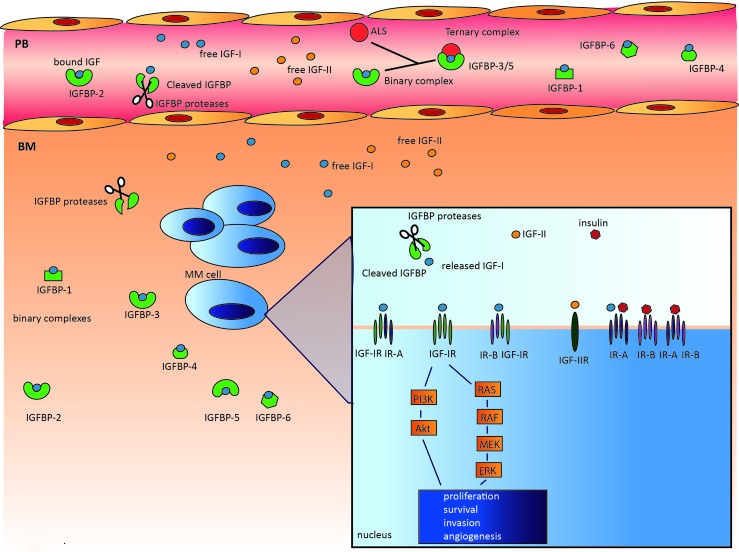
The complexity of the IGF system The IGF system is a highly complex system composed of: IGF ligands (insulin, IGF-I, IGF-II), cell surface receptors (IR, IGF-IR, IGF-IIR, and IGF-1R/IR hybrids), a family of six high-affinity IGFBPs, and several IGFBP proteases, including serine proteinases, aspartic acid proteases, and metalloproteinases. In the circulation, IGFs are mostly bound to IGFBPs, greatly prolonging their half-life. In addition, binary complexes with IGFBP-3 and IGFBP-5 can form a 150 kDa ternary complex with the acid-labile subunit (ALS). In contrast to the binary complexes, these ternary complexes are unable to cross the endothelium. Within the BM microenvironment, the IGFBPs in the binary complexes may be cleaved by the abundantly available IGFBP proteases, thereby releasing the IGFs and increasing IGF bioavailability. The free IGF ligands will then bind the transmembrane receptors and activate two main signaling pathways, PI3K/Akt and MEK/ERK. Activation of these pathways results in proliferation, survival, invasion, and angiogenesis.

The IGF system is a key regulator of growth and energy metabolism and is composed of the following components: IGF ligands (insulin, IGF-I, IGF-II), cell surface receptors (insulin receptor (IR), IGF-IR, IGF-IIR, and IGF-IR/IR hybrids), a family of six high-affinity IGF binding proteins (IGFBPs), and IGFBP degrading enzymes collectively called IGFBP proteases. The system also includes proteins involved in intracellular signaling, such as Akt, Shc/Grb2, and the insulin receptor substrate (IRS) protein family (Figure 1) [35].

### IGF ligands

IGF-I and IGF-II are approximately 7 kDa peptides that share obvious sequence similarity with insulin [36, 37]. IGFs play a crucial role in normal growth and development in both mice and humans. IGF-I and IGF-II knockout (KO) mice exhibit marked prenatal and neonatal growth retardation. In addition, IGF-I KO mice also show postnatal growth retardation and severely reduced body weights dying soon after birth. Therefore, in mice, though both IGFs are important for prenatal and neonatal growth and development, IGF-I is primarily involved in postnatal growth [38]. In humans, IGF-I levels increase from birth until puberty and are known to be essential for longitudinal bone growth [39]. However, IGF-I levels start to decline with increasing age, and this is associated with bone loss [40]. In the MrOS Sweden Study, researchers demonstrated that older men with low serum IGF-I levels have an increased fracture risk due to bone loss [41]. The main source of postnatal IGF-I and IGF-II is the liver. IGF-I is produced systemically after the secretion of growth hormone (GH) into the bloodstream by the pituitary gland, whereas the IGF-II regulatory mechanisms remain unclear [42]. IGFs are also abundantly synthesized in local tissues through autocrine and paracrine mechanisms [43]. Transgenic mice have been used to investigate the local paracrine and autocrine actions of IGF-I [44]. In mice overexpressing IGF-I in specific organs and/or tissues, local overgrowth occurs without differences in the levels of circulating IGF-I. For example, overexpression of IGF-I in osteoblasts was reported to increase trabecular bone volume [45]. Moreover, an increase in brain size was reported in mice overexpressing IGF-I in the brain, as evidenced by an increase in the number of neurons [46, 47]. In addition, numerous studies have shown that IGF-I is a potent mitogen for a variety of cells by increasing DNA synthesis and stimulating the expression of cyclin D1, leading to the progression of cells from G1 to S phase [48]. In addition to roles in growth-related processes, IGFs have also been demonstrated to mediate cell migration and prevent apoptosis by stimulating the expression of anti-apoptotic Bcl proteins [49, 50].

### IGF-R

The cellular responses to IGFs are primarily mediated by the IGF-IR. This cell membrane receptor binds with higher affinity to IGF-I than IGF-II. The IGF-IR is expressed on different cell types and is a heterotetramer composed of two extracellular α-subunits and two transmembrane β-subunits bound together with disulfide bridges [51]. The IGF-IR closely resembles the IR, with 60% sequence identity [52]. The IR binds to insulin with higher affinity then IGFs and is considered an essential regulator of metabolism, specifically glucose transport and the synthesis of fat and protein. In contrast, the IGF-IR has a higher affinity for IGF-I and plays a major role in both cell and whole body growth. Due to alternative splicing of exon 11 in the IR gene, two isoforms exist: IR-A and IR-B. IR-A is ubiquitously expressed and seems to be involved mainly in the mitogenic and anti-apoptotic effects of insulin and IGF-II. In contrast, IR-B expression is limited to metabolic tissues and is only activated by insulin, which explains its metabolic function [53, 54]. Hybrid receptors composed of a half IGF-IR and half IR (either IR-A or IR-B depending on availability) can also form. These hybrids retain high affinity for the IGFs, and a lower affinity for insulin, and are often abundantly expressed in tumor cells. The extracellular part of the IR, IGF-IR, or IGF-IR/IR hybrid binds the ligand, and the intracellular part contains the tyrosine kinase activity [55]. Upon binding of the free ligand, the tyrosine kinase activity is activated, resulting in autophosphorylation of the receptor and the recruitment of adaptor molecules (e.g., IRS and Grb) that activate downstream pathways, such as PI3K/Akt and RAS/RAF/MEK-ERK. The PI3K/Akt pathway mainly mediates the anti-apoptotic effects of the IGF ligands, whereas the MEK-ERK pathway is mainly involved in regulating the cell cycle and proliferation. In addition to these main pathways, IGF-IR is able to activate the JAK/STAT pathway and Wnt and NF-κB signaling [56].

IGF-II has its own specific receptor, the IGF-II receptor (IGF-IIR), also known as the mannose-6-phosphate receptor. In contrast to the IGF-IR and IR, the IGFR-IIR is not a tyrosine kinase, but clears IGF-II from the circulation by internalizing and degrading cell surface attached IGF-II [57]. The IGF-IIR has only low binding affinity for IGF-I and insulin.

### IGFBPs and IGFBP proteases

The IGFBPs are a family of six proteins (24 to 35 kDa) with distinct functional properties but sharing high homology [58]. Although IGFBPs are mainly produced within the liver, many normal and malignant tissues also express IGFBPs and they can be found in various biological fluids [39]. In the circulation, all six IGFBPs bind with high affinity and specificity to the IGF ligands, forming binary complexes and serving as IGF carriers [59]. In addition, together with acid-labile subunit (ALS) protein, IGF-IGFBP-3 and IGF-IGFBP-5 form high molecular weight ternary complexes [60]. These complexes protect the IGF ligands from degradation and greatly prolong the circulating half-life of the IGFs, from a few minutes for the ‘free’ peptides to 16 hours or longer for the ternary complexes [61, 62]. Most of the circulating IGFs predominantly exist as ternary complexes with IGFBP-3. These ternary complexes are thought to be unable to cross the endothelium [62]. However, the binary IGF-IGFBP complexes are able to enter the target tissue [63]. In the target tissues, IGFBPs have complex and multiple functions, which can be either IGF-dependent or IGF-independent, as previously summarized by Baxter *et al.* and Conover *et al.* [59, 64]. IGFBPs can function as inhibitors of IGF-I and IGF-II activity by binding to the IGFs with a higher affinity than the IGF-IR (acting as an IGF carrier), thereby reducing IGF bioavailability. However, IGFBPs can also serve as reservoirs that slowly release the IGFs, increasing or prolonging IGF-IR signaling. Several reports have also described the IGF-independent effects of IGFBPs [58, 64]. For example, IGFBP-5 has been shown to stimulate bone formation *in vitro* and *in vivo* without interacting with IGF-I or IGF-IR [65]. In addition, IGFBP-2 binds to α5-integrins and is involved in glioma cell migration and invasion [66]. IGFBPs also regulate cell growth and survival by interacting with several signals, such as TGF-β and p53 [67, 68]. Intranuclear IGFBPs have been suggested to play a role in transcriptional regulation, the induction of apoptosis, and DNA damage repair. Furthermore, 10 IGFBP-related proteins (IGFBP-rP) have been described. These proteins have low affinity for the IGF ligands. Thus, the biological functions of the IGFBP-rPs are thought to be mainly IGF-independent and fall beyond the scope of this review [58].

The activity of the IGFBPs largely depends on posttranslational modifications, such as proteolysis and phosphorylation. Cleavage of IGFBPs by proteases results in increased free IGF-I bioavailability [59]. Proteases capable of cleaving IGFBPs have been identified within three major protease classes: serine proteinases, aspartic acid proteases, and metalloproteinases. For example, prostate-specific antigen (PSA), a serine proteinase, is able to cleave IGFBP-3 and IGFBP-5 [69], whereas cathepsin D, an aspartic acid protease, has been demonstrated to proteolyze IGFBP-1 through IGFBP-5 [70–72]. Metalloproteinases are also involved in the cleavage of IGFBP-2, IGFBP-3, IGFBP-4, and IGFBP-5 [73]. The zinc metalloproteinase, pregnancy-associated plasma protein-A (PAPP-A), has been demonstrated to be a local regulator of IGF bioavailability through cleavage of IGFBPs [74]. Phosphorylation can also regulate IGFBP activity. Serine phosphorylation of IGFBP-1 enhances the affinity for IGF-I by 6- to 8-fold, and its capacity to inhibit IGF-I actions [75]. IGFBP-3 and IGFBP-5 also undergo phosphorylation, thereby enhancing their IGF-I binding capacity [76]. Finally, the binding affinity of IGFBPs for the IGFs can be affected by the binding of IGFBPs to the cell membrane or extracellular matrix (ECM) [77].

## THE IGF SYSTEM IN MM

**Figure 2:**
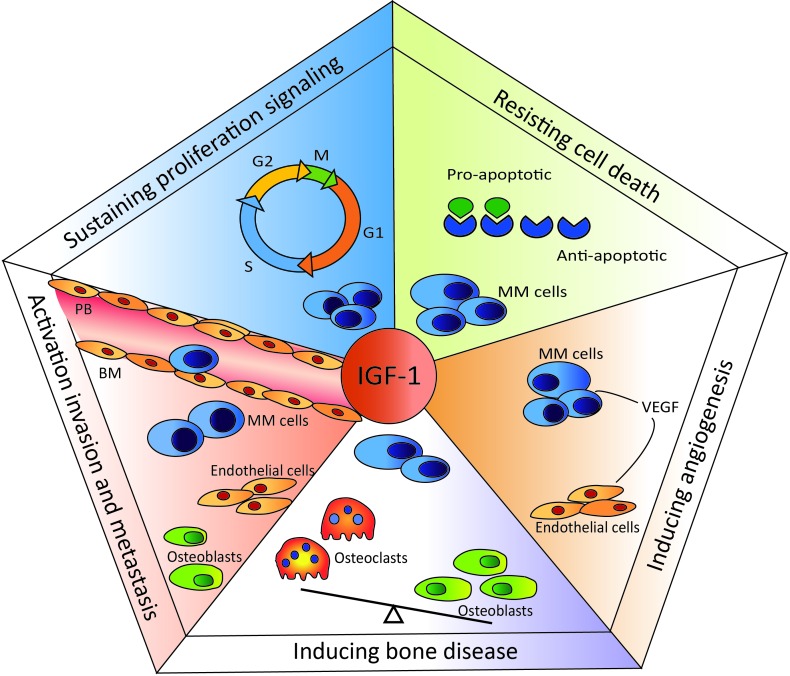
Role of IGF-I in multiple myeloma-specific hallmarks IGF-I is involved in the homing process, attracting the MM cells from the peripheral blood (PB) into the BM microenvironment. Once in the BM, IGF-I stimulates the proliferation of MM cells. Moreover, IGF-I up- and down-regulates the expression of anti- and pro-apoptotic molecules, respectively, protecting MM cells from (drug-induced) apoptosis. Another important hallmark of MM is the induction of angiogenesis. IGF-I stimulates VEGF secretion by the MM cells, enhancing angiogenesis. IGF-I is also involved in myeloma-associated bone disease by promoting osteoclast maturation and activity.

In 2000, Hanahan and Weinberg introduced “hallmarks of cancer”, proposing that the complexity of cancer can be reduced to six underlying mechanisms [78]. The key hallmarks of malignancy include sustained proliferative signaling, evasion of growth suppressors, resistance to cell death, replicative immortality, the induction of angiogenesis, and activation of invasion and metastasis. In 2011 two new hallmarks were proposed: abnormal metabolic pathways and evasion of the immune system [79]. Dysregulation of the IGF system has been demonstrated to support cancer development and progression in various cancer types, including prostate, breast, lung, colon, and different hematological cancers, by fostering most, if not all, of the key hallmarks [80, 81]. Below we summarize the role of the IGF system in the pathogenesis of MM, focusing on the most prominent key hallmarks of this specific malignancy: cell homing and invasion, sustained proliferation, resistance to cell death, and angiogenesis (Figure 2).

### Homing/invasion

Terminally differentiated monoclonal malignant PCs home to the BM microenvironment from either an extramedullary source or anatomically distant BM location. In both cases, the homing process is similar to that of lymphocytes and involves different consecutive steps: attraction of the MM cells to the BM, adhesion to the endothelium, and invasion through the endothelial layer and basement membrane into the ECM of the BM [82]. IGF-I is strongly involved in this homing process. We showed that IGF-I serves as a chemo-attractant for murine 5T33MM cells *in vitro* by activating the PI3K pathway [83]. We also demonstrated that the IGF-IR is upregulated in MM cells after interaction with the BM niche [84]. This was confirmed by Tai *et al*. and Qiang *et al*., who showed that IGF-I increases the adhesion of human MM cells via ß1-integrin and the PI3K pathway, and induced the migration and invasion of human MM cells [85, 86]. Furthermore, the α4ß1 integrin inhibitor natalizumab has been shown to inhibit IGF-I-mediated MM cell migration on fibronectin [87]. In a recent study, IGF-I was demonstrated to synergize with SDF-1 in promoting the migration of MM cells, further confirming its major role in the homing process [88]. In addition, Pichiorri *et al*. demonstrated that miR-192 and miR-215 prevent the migration of malignant PCs into the BM via indirect targeting of IGF-IR [89]. miR-192 and miR-215 regulate the expression of murine double minute 2 (MDM2), a negative regulator of p53 [90]. MDM2 serves as a ligase in ubiquitination of the IGF-IR, causing its degradation [91]. Taken together, these results clearly demonstrate that IGF-I is an important migration factor in MM. As mentioned earlier, IGF-I bioavailability in the tissues is mainly regulated by IGFBPs and IGFBP proteases. Previously, we and others demonstrated that matrix metalloproteinases (MMPs) produced by MM cells play a major role in the homing process [92–94]. In short, we demonstrated for both human MM cells and murine 5TMM cells that interaction with the BM endothelium enhances the secretion of MMP-9 by the MM cells, increasing MM cell invasion [93, 94]. Therefore, one could speculate that MMPs may promote homing of the MM cells by increasing IGF-I bioavailability. However, the bioavailability of IGF-I in relation to the activity of MMPs in the BM niche has not yet been investigated in MM and warrants future investigation.

After the homing of MM cells to the BM they reside in the BM microenvironment, where they interact with the different BM stromal cell (BMSC) types. The BM microenvironment consists of hematopoietic and non-hematopoietic cells. The hematopoietic cells consist of myeloid (e.g., erythrocytes, macrophages) and lymphoid (e.g. T cells, B cells) cells, whereas the non-hematopoietic cells include BM fibroblasts, BM endothelial cells (BMECs), osteoclasts (OCs), osteoblasts (OBs), adipocytes, and mesenchymal stem cells. MM cells interact with BMSCs mainly through adhesion molecules and cytokines. Along with IL-6, IGF-I is one of the most important growth factors in MM [30]. IGF-I is abundantly available in the BM microenvironment and is produced by both BM fibroblasts and OBs [95].

### Proliferation

The first evidence of IGF-I as a mitogenic factor for MM cells came from Freund *et al*. [31], but numerous studies have confirmed the mitogenic activity of IGF-I in MM. Georgii-Hemming *et al*. demonstrated that IGF-I can act as a growth factor for human MM cell lines and reported that an autocrine IGF-I loop may contribute to the growth and survival of MM cells [96]. In addition, IGF-I has been reported to enhance IL-6 activity and induce the proliferation of both IL-6-dependent and IL-6-independent cell lines [97, 98]. Later on, Sprynski *et al*. showed that only a subpopulation (CD45-) of human MM cell lines can survive through an autocrine IGF-I/IGF-IR loop. In addition, they found that the increased proliferation of human MM cell lines induced by IL-6 is dependent on the presence of an autocrine IGF-I loop [30]. Furthermore, autocrine IGF-I, but not IL-6, was recently described as being the main self-clonogenic growth factor for myeloma cell lines [99]. Finally, Huang *et al*. found that EEN, which regulates endocytosis, also regulates the proliferation and survival of MM cells by regulating IGF-I secretion [100]. IGF-I was shown to be able to reverse the inhibition of proliferation caused by the knockdown of EEN. In contrast, an IGF-I targeting antibody inhibited the proliferative effect of EEN overexpression. Unexpectedly, insulin is also a potent growth and survival factor for MM cell lines and primary MM cells. This growth-stimulating effect has been shown to be mediated through IS/IGF-I hybrid receptor activation [101].

### Cell death

Next to it, IGF-I also promotes the cell survival of serum-starved MM cells [85, 97, 102]. The balance of pro- and anti-apoptotic molecules determines whether a cell will undergo apoptosis [103]. In MM, the upregulation of anti-apoptotic molecules is associated with increased MM cell survival and chemoresistance [104]. IGF-I treatment has been demonstrated to upregulate Fas apoptosis inhibitory molecule (FAIM) expression in MM cells, leading to increased MM cell survival [105]. In addition, we demonstrated that IGF-I downregulates the mRNA and protein expression of the pro-apoptotic molecule Bim in human MM cell lines and murine 5T33MM cells [106]. Moreover, preclinical studies have provided strong evidence that IGF-I protects against various standard and novel anti-MM agents, such as dexamethasone, Apo2/TRAIL, ABT-737, NF-κB inhibitor, and bortezomib [107–110]. Concerning ABT-737, our group demonstrated a clear synergistic anti-myeloma effect of the IGF-IR inhibitor picropodophyllin and ABT-737 [107]. In addition, in contrast to what we would expect, lenalidomide has been shown to enhance *IGF-I* mRNA levels in almost all human cell lines and primary myeloma cells tested [111]. Consequently, the IGF-I/IGF-IR loop may be involved in the progression of secondary cancers, a strong contraindication observed after long-term treatment of MM patients with lenalidomide [112].

### Angiogenesis

We and others have also shown that IGF-I stimulates MM cells to produce VEGF through the MEK/ERK pathway, leading to increased angiogenesis in the BM [113, 114]. Angiogenesis is a process of new blood vessel formation from the pre-existing vasculature and plays an essential role in development, reproduction, and repair [115]. It is a multi-step process including endothelial cell (EC) activation, degradation of the ECM components, EC migration, and organization of ECs to form blood vessels. The formation of blood vessels involves a complex interplay between positive or pro-angiogenic factors (e.g., VEGF, angiopoietins, members of the fibroblast growth factor (FGF) family, and MMPs) and negative or anti-angiogenic factors (e.g. endostatin, angiostatin, IL-12, thrombospondin) controlling the survival, growth, migration, and MMP secretion of ECs. An imbalance in the levels of these pro- and anti-angiogenic factors contributes to the pathogenesis of different disorders, including cancer [116]. Angiogenesis has been demonstrated to be essential for tumor growth, invasion, and metastasis in various cancer types [117]. In MM, angiogenesis has been shown to strongly increase from MGUS to MM due to a shift in the pro- and anti-angiogenic balance towards angiogenesis (angiogenic switch) [118–120]. Enhanced BM angiogenesis in MM patients (as measured by the microvessel density) has been demonstrated to correlate with poor prognosis [121, 122]. VEGF is one of the main pro-angiogenic factors in MM and is produced by MM cells and BMSCs. Additional pro-angiogenic factors have also been described in MM, including bFGF, hepatocyte growth factor (HGF), and angiotensin 1 (Ang-1) [123]. bFGF is able to enhance angiogenesis by inducing EC activation [124]. Ang-1 is produced by the MM cells and upregulates the Ang-1 receptor Tie2 in human BM ECs, increasing their angiogenic potential [125]. MMPs are also involved in the angiogenic process in MM by degrading the ECM [126, 127]. Using 5TMM murine models and human MM cell lines, we have shown that targeting the IGF-IR inhibits angiogenesis both *in vitro* and *in vivo* [128]. However, one study failed to show a correlation between serum IGF-I levels and angiogenic cytokines in MM patients [129], as IGF-I serum levels were found similar in MM patients and healthy controls.

### Bone disease

Another important clinical hallmark of MM is the occurrence of bone lesions. Approximately 90% of MM patients have bone lesions, and 80% of MM patients encounter bone fractures during the course of their disease [130, 131]. Recently, Farr *et al*. reported that even MGUS patients already have an increased fracture risk compared to healthy controls, as they were found to have increased bone porosity and reduced bone strength [132, 133]. The bone destruction is the result of an imbalance in bone formation and resorption due to an increase in the number and activity of OCs and suppressed OB formation and functionality. Multiple factors that increase OC activity (OC inducers) have been identified in MM, including receptor activator of nuclear factor (NF)-κB ligand (RANKL), macrophage inflammatory protein (MIP)-1α, TNF-α, IL-6, IL-3, and IL-1. The binding of RANKL to RANK receptor triggers OC activation and leads to increased bone resorption [134]. This bone resorption is counteracted by osteoprotegerin (OPG), which binds to RANKL and prevents it from interacting with RANK [135]. In MM patients, however, RANKL is overexpressed as OPG levels decrease, leading to a disturbed RANKL/OPG balance favoring bone resorption [136]. MM cells also produce MIP-1α, and increased MIP-1α serum levels correlate with lytic bone lesions in MM patients [137, 138]. In addition, the MM cells express and secrete factors that are able to suppress OB differentiation and activity. An important regulator of OB formation is the Wnt signaling pathway, and a major inhibitor of OB differentiation is the Wnt signaling antagonist Dickkopf (DKK-1). DKK-1 levels are highly elevated in MM patients with osteolytic lesions [139]. In addition, secreted Frizzled-related protein-2 (sFRP-2), another Wnt signaling antagonist, has been demonstrated to suppress bone formation in MM [140]. Furthermore, the transcription factor Runt-related transcription factor2 (Runx2) has been demonstrated to be involved in suppressing OB formation and to enhance tumor growth and disease progression [141]. Although the IGF system is known to play a crucial role in bone metabolism, its exact role in MM bone disease is mostly unknown [142]. In 1999, Feliers *et al.* reported that human MM cells express *IGFBP-4*, and to a lesser extent *IGFBP-6* [143]. They demonstrated an accumulation of IGFBP-4 within the BM microenvironment and suggested that this may lead to a suppression of OB formation due to a decrease in bioavailable IGF-I. In contrast, we showed that picropodophyllin reduces the number of osteolytic lesions and OCs in MM-bearing mice and decreases the *in vitro* bone resorption activity of the OCs [144]. More recently, the glycosphingolipid GM3 produced by MM cell lines and primary MM cells was demonstrated to cooperate with IGF-I and RANKL to promote OC maturation [145]. Taken together, the data clearly suggest a direct role of IGF-I in MM bone disease [144].

**Figure 3:**
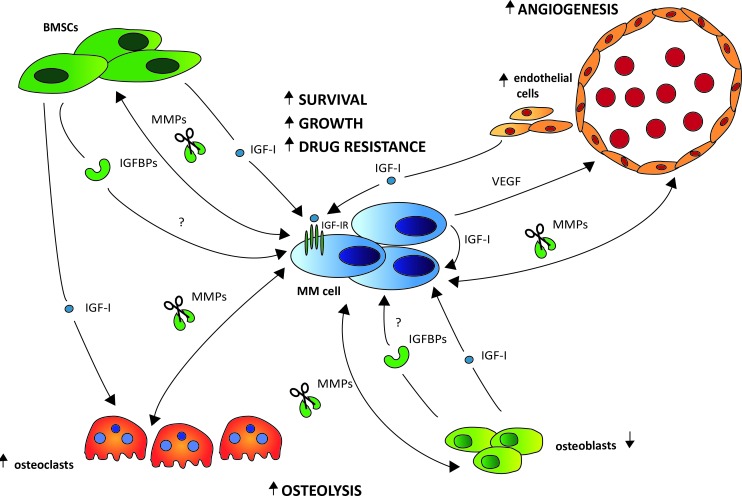
The role of the IGF system in the bi-directional interactions of the MM cells and the different stromal cell types Upon interaction with the MM cells through adhesion molecules, IGF-I is abundantly produced by different BM stromal cell types, including the BM fibroblasts (BMSCs), osteoclasts (OCs), osteoblasts (OBs) and endothelial cells (ECs). Binding of IGF-I to the IGF-IR expressed on the surface of the MM cells will then foster MM cell survival, growth and drug resistance. The increased IGF-I secretion will also stimulate the MM cells to produce VEGF, leading to enhanced BM angiogenesis further fostering MM cell survival and growth. Moreover, IGF-I will also promote bone destruction by enhancing OC migration and activation. This bone destruction will in turn result in the release of numerous growth and survival factors embedded in the bone matrix, thus further driving the vicious cycle. In the extracellular environment, the majority of IGF is known to be bound to one of six IGF-binding proteins (IGFBP1-6), leaving only a minor fraction of total IGF free and accessible for receptor activation. Although preliminary evidence indicates an abnormal IGFBP profile in MGUS and MM leading to an increased IGF-I bioavailability, the exact role of the IGFBPs in the pathobiology of MM and the major source in the BM niche remains to be investigated. Cleavage of the IGFBPs by protease, including matrix metalloproteinases (MMPs), also results in increased IGF-I bioavailability. In MM, interaction of the MM cells with the different stromal cell types strongly enhances the production of MMPs not only by the MM cells but also by the stromal cells.

Thus far, no or limited data is available on the role of IGF-I in the remaining key hallmarks of malignancy, namely replicative immortality, abnormal metabolic pathways, and evasion of the immune system. A schematic overview illustrating the role of the IGF system in the cross-talk between the MM cells and the relevant BM stromal cell types is provided in Figure 3.

## DIAGNOSTIC AND PROGNOSTIC POTENTIAL OF THE IGF SYSTEM IN MM

Given the important role of the IGF system in the pathogenesis of MM, several research groups have investigated the potential of using the different components of the IGF system as biomarkers of MM development and progression. The National Institutes of Health has defined a biomarker as “a characteristic that is objectively measured and evaluated as an indicator of normal biological processes, pathogenic processes, or pharmacologic responses to a therapeutic intervention” [146].

Reports regarding IGF-I serum levels and MM risk and prognosis have been contradictory. Standal *et al*. were the first to examine the relationships between the levels of circulating IGF and MM risk and prognosis. They found no differences in the total IGF-I levels in serum for 127 MM patients and 42 healthy controls but detected a strong correlation with survival within the MM group [147], as patients with low blood IGF-I levels had a more favorable prognosis. Similar results were found by Pappa *et al*., who did not show significant differences in serum IGF-I concentrations between MM patients and controls [129]. In contrast, Greco *et al*. observed significant, gradual reductions in the total IGF-I levels in MGUS and MM patients compared to healthy subjects [148]. These discrepancies may be due to methodological issues, such as the use of different IGF-I ELISA kits, some of which encounter analytical problems, or the lack of a consensus for the collection and storage of samples [149]. In addition, systemic levels of total IGF are unlikely to tell the whole story. First, by measuring total IGF-I levels, no discrimination is made between bound and free (bioavailable) IGF-I. Second, MM cells are capable of producing their own IGF-I and stimulate IGF-I secretion by BMSCs [120]. Therefore, circulating IGF levels are likely very different from the local IGF-I levels and probably not directly relevant. However, regardless of the explanation, the conflicting reports indicate that circulating IGF-I levels are not an ideal biomarker of MM risk and progression, and that the IGF-IR could be a better candidate.

Bataille *et al.* showed that IGF-IR is aberrantly expressed on MM cells and that increased expression is indicative of poor prognosis [150]. Using gene expression profiling data from two independent cohorts of previously untreated MM patients, increased *IGF-IR* mRNA expression was found to correlate with a poor prognosis [30]. However, Chng *et al.* failed to find a difference in *IGF-IR* mRNA expression between normal PCs and malignant PCs from MGUS, smoldering MM (SMM), and MM samples. Nevertheless, it was shown that the IGF-IR is overexpressed in the subtypes of MM with poor prognosis [151]. Taken together, the data demonstrate that higher IGF-IR expression is linked with a worse disease outcome in MM.

So far, very little work has addressed the diagnostic and prognostic potential of the IGFBPs in MM. Standal *et al.* found no significant difference in total IGFBP-3 levels in serum between MM and healthy patients [147]. However, despite IGFBP-3 being generally known as the most abundant IGFBP in the circulation, other IGFBPs could be important in MM disease. This was demonstrated by the peripheral blood levels of IGFBP-1 positively associating with an increased risk of progression from MGUS to MM within 3-6 years of blood collection [152]. Moreover, Brandt *et al.* purified IGFBP-1 protease activity from the urine of a MM patient, but no specific protease activity was detected within the serum of the patient. This could mean that the IGFBP complex remains intact in the circulation [153]. In addition, gene expression levels of the so-called IGFBP7 gene, also known as MAC25, Prostacyclin-Stimulating Factor, Tumor-Derived Adhesion Factor, or PGI2-Stimulating Factor, have been linked to poor prognosis [154]. IGFBP7 is not, however, a high affinity IGF binding protein and is not generally considered part of the extracellular IGF system [155]. Similar to IGF levels, the distribution of IGFBPs between the different fluids and tissues varies greatly [39]. Therefore, local IGFBP levels in the BM niche are likely to be more informative regarding IGF bioavailability and IGF signaling. Recently, we were the first to investigate IGFBPs in paired peripheral blood and BM plasma samples from MM, MGUS, and control individuals. We demonstrated a strong increase in circulating levels of IGFBP-2 in both MGUS and MM patients. Within the BM microenvironment, however, we demonstrated a reduction in the total IGFBP and IGFBP-2 levels in MGUS and MM patients compared to controls (IN PRESS in PLOS ONE). Therefore, MGUS and MM patients may have greater IGF bioavailability within the BM microenvironment.

Thus, despite the strong involvement of IGF system components in the pathogenesis of MM, its diagnostic and prognostic potential in MM remains largely inconclusive, and future studies are urgently needed to further investigate and compare the levels of free and bound IGF-I and IGFBPs in the circulation and BM niche during disease progression.

## THERAPEUTIC POTENTIAL OF TARGETING THE IGF SYSTEM IN MM

With the large body of preclinical proof that the IGF-I/IGF-IR axis plays a pivotal role in the pathogenesis of MM, interest in this axis as a potential therapeutic target has grown. Several strategies to specifically target the IGF-IR have been developed and tested for their anti-myeloma activity in preclinical myeloma models and clinical trials [80, 156].

### Preclinical myeloma models

Over 100 human MM cell lines have been generated from patients with advanced disease, obtained mostly from extramedullary sites [157]. The characteristics of approximately half of these cell lines were summarized and classified into different molecular subgroups using gene expression profiling [158]. Although a lot of information can be extracted from *in vitro* tests with human MM cell lines and primary MM cells, these models fail to recapitulate the complex, bidirectional interactions with the BM niche. Moreover, in the context of the IGF-I system it is worth mentioning that MM cell lines are usually generated in the presence of serum containing high levels of IGF-I. This makes the MM cell lines to a certain extent prone to IGF-I and might thus create a certain bias. Therefore, preclinical *in vivo* MM models are crucial for testing the anti-myeloma activity of novel therapeutic agents. Different preclinical *in vivo* models of MM have been developed, each with their own specific advantages and disadvantages [159, 160]. These models can be subdivided into i) xenograft models of human MM cells into mice (e.g., SCID-hu, NOD/SCID, and SCID-Rab models), ii) models of murine MM (e.g., 5TMM models and transgenic models), and iii) pristane-induced plasmacytoma in BALB/c mice [161].

The most widely used preclinical MM models for testing the anti-myeloma activity of (new) therapeutic agents *in vivo* are the severe combined immunodeficient (SCID) xenograft models. In these models, human MM cells (either primary MM cells or human MM cell lines) are engrafted in different ways into immunodeficient mice, giving rise to several variants, including the (NOD)/SCID, SCID-hu, and SCID-synth-hu xenograft models. However, the major limitations of all of these xenograft models are the lack of a functional immune system and a fully compatible microenvironment [162, 163].

The 5TMM models are a series of syngeneic and immunocompetent murine MM models representing various *de novo* MM variants, ranging from smoldering to highly aggressive MM. The 5TMM models originated spontaneously in elderly C57BL/KaLwRij mice and were propagated *in vivo* by intravenous injection of diseased BM into young syngeneic animals [160, 164]. These models resemble the human disease at both the molecular and cellular level, with localization of the MM cells in the BM, presence of an elevated serum M-spike correlating with disease progression, and the development of MM-associated angiogenesis and bone disease [160]. As such, the models are highly suited to studying the bidirectional interactions between MM cells and the microenvironment (including the immune system) and for preclinical testing of novel agents targeting MM cells and/or the BM niche [164]. The best characterized and most widely used 5TMM models are the 5T2MM, 5T33MM, and 5TGM1 models (the latter being a variant of the 5T33MM model). The 5T2MM model represents a moderately growing MM, with full blown MM in ~10 to 12 weeks, and exhibits marked MM-associated bone disease. In contrast, the 5T33MM and 5TGM1 models are more aggressive forms of the disease, developing MM after 4 to 6 weeks [160, 165].

In addition to the 5TMM models, genetically engineered murine models have also been developed, such as the Vk*MYC model [166]. In this transgenic mouse model, the Vk*MYC transgene is introduced into the C57/BL6 mouse strain that spontaneously develops a high rate of monoclonal gammopathy, resulting in fast MM progression. The Vk*MYC model has strong homology with the human disease with similar clinical and biological characteristics. In addition, with congenic transplantation of Vk*MYC tumor cells, the model can also be used as a model of relapsed human MM. Thus, the Vk*MYC model is suited to studying MM biology and has been demonstrated to predict the efficacy of drugs in both untreated and relapsed patients [167]. Other examples of transgenic models are the Eμ-XBP1s and Eμ-MAF models deregulating the XBP1s and MAF oncogene, respectively [168]. However, the use of these models is rather limited, as maintenance is labor-intensive and time consuming. In addition, there is often a long and heterogeneous lag time to tumor development, making it difficult to study tumor progression in a large cohort of mice [169].

Although mice are the most common model organism for preclinical studies, there are well-known quantitative and qualitative differences between mice and humans. Therefore, the transferability of data generated in mice to humans could be questioned [170, 171]. To overcome this issue, it has been suggested to use whole bone marrow aspirates or ex vivo 3D models. Concerning the latter, Ferrarini et al. developed an innovative 3D dynamic culture model, which allows ex-vivo culturing of primary human MM cells inside their native microenvironment with maintenance of the original tissue architecture (including vessels and bone lamellae). This model was shown to be an excellent tool to predict response to bortezomib in MM patients. It was demonstrated that the expression levels or activity of surrogate markers of specific functions of both MM cells and the microenvironment (including ß2 microglobulin, VEGF, angiopoietin-2 and MMPs) in the supernatants, could predicted the response to bortezomib treatment [172].

### IGF therapies

Numerous therapeutic agents that selectively target the IGF-I/IGF-IR axis have been developed and can be roughly divided into three general categories: monoclonal antibodies targeting the IGF-IR, small molecule tyrosine kinase inhibitors (TKIs), and IGF-I-targeting antibodies [173]. IGF-IR monoclonal antibodies prevent binding of the ligand to the receptor and induce receptor internalization and degradation, whereas TKIs decrease receptor activity by competing with ATP for binding the kinase domain and IGF monoclonal antibodies prevent ligand binding and activation of the receptor by neutralizing IGFs. Many of these agents have been preclinically tested in MM with very encouraging results [114, 144, 174]. Among the numerous other cancer cell lines, human MM cell lines are the most sensitive for IGF-IR inhibition [175].

Examples of IGF-IR monoclonal antibodies that have been tested in MM and demonstrated to have potent single agent activity in several preclinical models are CP-751,781, AVE1642, and A12 [32, 176, 177]. Descamps *et al*. also demonstrated *in vitro* synergistic effects for bortezomib and AVE1642 using human MM cell lines [32]. NVP-ADW742, NVP-AEW541, and picropodophyllin are TKIs that selectively target the IGF-IR [178]. NVP-ADW742 and NVP-AWE541 are both ATP-competitive inhibitors reported to potently block the survival and growth of MM cells (both human cell lines and primary MM cells), potentiating the anti-myeloma activity of standard-of–care agents, including melphalan, dexamethasone, doxorubicin, bortezomib, and lenalidomide, and prolonging the survival of orthotopic xenograft MM models [114, 178]. Picropodophyllin, a non-ATP-competitive IGF-IR TKI, has also been shown to block autophosphorylation of the IGF-IR, thereby inhibiting growth and inducing apoptosis in human MM cell lines and primary MM cells [179]. In addition, we demonstrated that picropodophyllin significantly reduces tumor burden and prolongs the overall survival of 5T33MM-inoculated mice without observing any major IR-related toxicities [128]. We also confirmed this potent *in vivo* anti-myeloma activity in a therapeutic setting using the 5T2MM model. Continuous treatment after the onset of disease strongly reduced the tumor burden and extensively prolonged survival [144]. Furthermore, we demonstrated that picropodophyllin inhibits MM-associated angiogenesis and bone disease [128, 144]. Nevertheless, picropodophyllin-treated mice eventually relapse and exhibit signs of morbidity. Therefore, we performed high throughput screenings to select agents that are able to increase the anti-myeloma activity of picropodophyllin. One class of drugs coming out of these high throughput screenings are the histone deacetylase inhibitors (HDACi). In line with this, the HDACi panobinostat was found to strongly enhance the anti-myeloma activity of picropodophyllin both *in vitro* and *in vivo* by down-regulating cell cycle and anti-apoptotic proteins [180]. In addition, our group showed that IGF-I protects human MM cell lines against ABT-737-induced cell death, and we hypothesized that IGF-IR targeting may overcome this resistance. Using human MM cell lines and primary MM cells, we observed a strong synergistic anti-myeloma effect when MM cells were co-treated with picropodophyllin and the Bcl-2 homology domain 3 (BH3) mimetics ABT-737 and ABT-199 [107]. Mechanistically, we showed that silencing of Bcl-2 abrogated ABT-737-induced lethality, whereas co-treatment with picropodophyllin was able to overcome this Bcl-2 dependency. In addition, in the 5T33 model, the combination significantly decreased tumor burden and prolonged the overall survival of the mice. Furthermore, we showed that the combination targeted the CD138+ and CD138- MM subpopulations equally. We and others previously showed that CD138- MM cells are more resistant to most standard-of-care agents, including PIs [181, 182]. IGF-I was also shown to be involved in bortezomib resistance. Targeting the IGF-IR using picropodophyllin or the clinically relevant dual IGF-IR and IR TKI OSI-906 overcame this resistance both *in vitro* and *in vivo* [108]. Finally, Liang *et al.* reported potent preclinical anti-myeloma activity (both as a single agent and when combined with lenalidomide or dexamethasone) for yet another novel small-molecule inhibitor of IGF-IR and IR, GTx-134 [174].

Despite the encouraging preclinical data, only a few clinical trials have been conducted in MM, thus far with largely disappointing results. In a phase I trial of relapsed MM, CP-751,871 was demonstrated to be safe and well-tolerated when used as a single agent, but no clear responses were observed. However, in combination with dexamethasone, 9 of the 27 patients experienced objective responses without observing any dose-limiting toxicities [183]. Moreau *et al*. tested AVE1642 both as a single agent and in combination with bortezomib in patients with relapsed MM. Although the drug(s) had a good toxicity profile, the response rates were considered insufficient to continue with the development of this molecule [34]. A phase I dose escalation trial was also started with MK-0646 (dalotuzumab) in advanced MM [184]. Dalotuzumab is a humanized IgG1 monoclonal antibody against the IGF-IR and acts by inhibiting IGF-I- and IGF-II-induced tumor cell proliferation and IGF-IR autophosphorylation [185]. Although this trial was recently completed, no results have yet been published. Based on the observation that OSI-906 resensitizes MM cells to bortezomib, patients are currently being enrolled in a new phase I/II trial testing OSI-906 in combination with bortezomib for the treatment of relapsed MM [108, 184].

## LESSONS LEARNED FROM PREVIOUS CLINICAL TRIALS

The preclinical evidence for the role of the IGF system in cancer is impressive, but unfortunately has failed to be translated into new anti-cancer therapies [186]. Although results obtained from preclinical studies and early clinical trials were very promising, most large randomized phase II and III trials have been largely disappointing in most cancer types, including MM. Consequently, many drug development programs targeting the IGF-IR have been discontinued. Several possible explanations have been suggested for the failure of IGF-IR therapy, as reviewed in [187–189]. Firstly, most past clinical trials failed to incorporate the use of predictive biomarkers for the selection of probable responders. Currently, no consensus is yet available on which biomarkers to use for selecting the ideal patient population. Several preclinical studies and retrospective analyses of patient materials for candidate biomarkers are ongoing. These candidate biomarkers can include all members of the complex IGF system (both systemic and local/tumor expression levels) and markers connected to the IGF system, including the downstream signaling pathways and gene signatures indicative of increased tumor insulin/IGF-I activity. Concerning the insulin/IGF system biomarkers, systemic insulin levels and tumor IR or IGF-IR/IR hybrid expression levels have been suggested to be more predictive than the IGF and IGF-IR homodimer levels. For the markers connected to the IGF system, constitutive activation of the downstream PI3K/Akt and MEK-ERK pathways via activating mutations have been reported to be negative markers of drug sensitivity [190, 191].

Secondly, most early trials, including in MM, neglected to determine the optimal timing of administration and/or optimal drug combinations. In these trials, IGF-IR targeting agents were tested mostly in heavily pre-treated relapsed/refractory patients. However, increasing evidence indicates that previous treatment could influence the dependency of the tumor cells on the insulin/IGF system and/or downstream signaling pathways. In myeloma, IGF-I was shown to be involved in bortezomib resistance, and insulin/IGF-IR targeting was shown to resensitize the MM cells [108]. Moreover, last year Frassanito et al. demonstrated that BM fibroblasts or cancer-associated fibroblasts (CAFs) from bortezomib resistant patients produce high levels of different growth factors (including IGF-I and TGFß) and protect the MM cells from bortezomib induced apoptosis [192]. Therefore, it may be beneficial to combine PIs and insulin/IGF-IR targeting agents in upfront treatment. In various cancers, IR/IGF-IR signaling has also been demonstrated to protect cells from DNA damaging agents by activating DNA double-strand break repair, and IGF-IR targeting has been shown to sensitize cells to doxorubicin, cisplatin, and ionizing radiation [193, 194]. The most effective anti-tumor activity has been observed when the IGF-IR targeting agent is administered after the DNA-damaging agent. In support of this, direct contact with BMSC was only very recently shown to counteract doxorubicin and 4-hydroxycyclophosphamide induced hypermethylation of H3K27 in MM cells via phosphorylation-mediated inactivation of EZH2, leading to the sustained expression of anti-apoptotic genes (including *IGF1*, B cell CLL/lymphoma 2 (*BCL2*), and hypoxia inducible factor 1α subunit (*HIF1A*)) and hence cell adhesion–mediated drug resistance (CAM-DR). Pharmacological and genetic inhibition of the IGF-IR/PI3K/AKT pathway reversed this CAM-DR [195]. Therefore, insulin/IGF-IR targeting agents may also be considered as consolidation/maintenance therapy in MM.

Thirdly, until now, IGF system targeting strategies have mainly focused on inhibiting the IGF-IR and have substantially neglected the true complexity of the IGF system. In this regard, all IGF ligands can activate IR-A hybrid receptors and IGF-IR monoclonal antibodies and most first-generation TKIs are unable to neutralize these hybrid receptors. Therefore, IGF-IR inhibitor resistance may be driven by IR/hybrid signaling. In addition, circulating IGF-IR can interact with the IGF-IR targeting antibodies and prevent their interaction with the IGF-IRs on cancer cells [196]; it was reported that only 20% of the administrated antibody will interact with the cancer cells. Combining IR-A/IGF-I tyrosine kinase inhibition with IGF-targeting monoclonal antibodies may overcome this problem. Also, as multilayered crosstalk is widely accepted to exist and constitutive activation of the PI3K/Akt and MEK-ERK pathways is a negative marker of drug sensitivity, the potential of inhibiting the PI3K/Akt and MEK-ERK pathways to overcome resistance to the insulin/IGF-I system inhibitors has become the subject of intense investigation. Moreover, the IGFBPs also play a prominent role in regulating the actions of IGFs. The development of mutant, protease-resistant IGFBPs may be a promising new approach to decreasing IGF bioavailability. Recently, protease-resistant and protease-resistant/non-matrix-binding variants of IGFBP-2 were designed and demonstrated to inhibit tumor growth by inhibiting angiogenesis [197]. The lack of protease and matrix-binding sites was suggested to block the ability of IGFBP-2 to promote both IGF-dependent actions (through the release of IGFs to the receptors) and IGF-independent actions (through ECM binding).

Lastly, drug-induced reduction of IGF-IR signaling interrupts the pituitary feedback loop regulating IGF-I production, leading to higher IGF-I levels and, indirectly, insulin levels [198]. Reduced IGF-IR signaling thus results in hyperglycemia, which is the most common toxicity [173]. Although this hyperglycemia is mild and reversible, it is well established that long-term hyperglycemia and hyperinsulinemia are important risk factors for cancer onset and progression in patients with obesity and/or type 2 diabetes. To limit these adverse effects and prevent treatment failure it can therefore be hypothesized that next to lifestyle changes insulin sensitizers (e.g. metformin) should be combined with the IGF system inhibitors [187]. In addition, more efforts should be made to identify more selective targets to improve tumor cell selectivity and avoid IGF system related toxicity. In this regard, studies are indicating that single nucleotide polymorphisms (SNPs) in IGF system related genes modify the activity/function of the IGF system. In addition, there is also evidence that IGF-I splice variants are differentially expressed between non-cancerous and cancerous cells, implying that the expression pattern of the various IGF-I transcripts and the respective proteins may have different functions in cancer biology [199]. Thus, future genome wide studies in MM should focus on the identification of genetic variants of the IGF system as a whole.

## CONCLUSION AND FUTURE PERSPECTIVES

The IGF system has been demonstrated to be involved in almost all of the MM-specific cancer hallmarks, including MM cell proliferation, survival, homing, and drug resistance, and MM-associated angiogenesis and bone disease (Figure 2). Despite its importance in the pathogenesis of MM, the therapeutic benefit of targeting the IGF system remains rather limited. Importantly, lessons learned from earlier clinical trials have taught us that much more preclinical research is needed to i) better understand the complexity of the IGF system, ii) identify more selective targets and iii) identify and validate candidate biomarker(s) using standardized protocols to stratify the patients. Given the system's complexity, it seems reasonable to assume that a combination of multiple factors will be required rather than one single predictive biomarker. In addition, a better understanding and characterization of the whole IGF system will help pave the way for identifying new, more selective targets and designing more rational combination therapy. Only then will we know if the renewed interest in the therapeutic potential of IGF-I targeting is justified.
